# Supplementary material for: Voltage-sensing phosphatase (Vsp) regulates endocytosis-dependent nutrient absorption in chordate enterocytes
Source: Commun Biol. 2022 Sep 10;5:948. doi: 10.1038/s42003-022-03916-6 (PMC9464190; doi:10.1038/s42003-022-03916-6)
Supplement: Supplementary file 2 — Supplementary Information [file 42003_2022_3916_MOESM2_ESM.pdf]

Supplementary Information

Voltage-sensing phosphatase (Vsp) regulates endocytosis-dependent nutrient absorption in chordate enterocytes

Adisorn Ratanayotha[1,2,3], Makoto Matsuda[1], Yukiko Kimura[4], Fumiko Takenaga[1], Tomoaki Mizuno[5], Md. Israil Hossain[1], Shin-ichi Higashijima[4], Takafumi Kawai[1*], Michio Ogasawara[6], Yasushi Okamura[1,7*]

Affiliations

[1] *Laboratory of Integrative Physiology, Department of Physiology, Graduate School of Medicine, Osaka University, Suita, Osaka, 565-0871, Japan*

[2] *Institute for Transdisciplinary Graduate Degree Programs, Osaka University, Suita, Osaka, 565-0871, Japan.*

[3] *Department of Anatomy, Faculty of Medicine Siriraj Hospital, Mahidol University, Bangkoknoi, Bangkok, 10700, Thailand*

[4] *Exploratory Research Center on Life and Living Systems and National Institute for Basic Biology, National Institutes of Natural Sciences, Okazaki, Aichi 444-8787, Japan*

[5] *Center for Medical Research and Education, Osaka University, Suita, Osaka, 565-0871, Japan*

[6] *Department of Biology, Graduate School of Science, Chiba University, Inage-ku, Chiba, 263-8522, Japan*

[7] *Graduate School of Frontier Biosciences, Osaka University, Suita, Osaka, 565-0871, Japan*

Correspondence

Yasushi Okamura (vsop1@me.com)

Takafumi Kawai (kawai@phys2.med.osaka-u.ac.jp)

Supplementary Information includes:

Supplementary Figures 1 – 9

Supplementary Tables 1 – 3

Supplementary References

Supplementary Figure 1

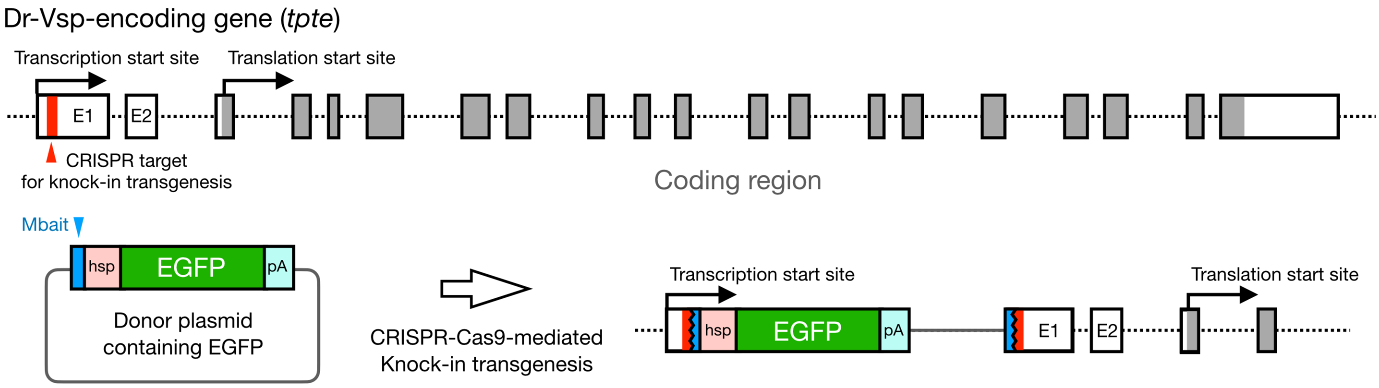

Supplementary Figure 1

Experimental schemes of CRISPR-Cas9-mediated *Tg(tpte:EGFP)* transgenesis

Generation of *Tg(tpte:EGFP)* transgenic zebrafish. Donor DNA plasmid containing the enhanced green fluorescence protein (EGFP) sequence was incorporated into *tpte* exon1, which is located between transcription start site (exon1) and translation start site (exon3). Successful transgenesis expresses EGFP recapitulating endogenous expression of *tpte* gene. For detailed information, please refer to the Methods section, and Kimura et al[1]. E1, exon 1. E2, exon2. hsp, hsp70 promoter. pA, polyA.

Supplementary Figure 2

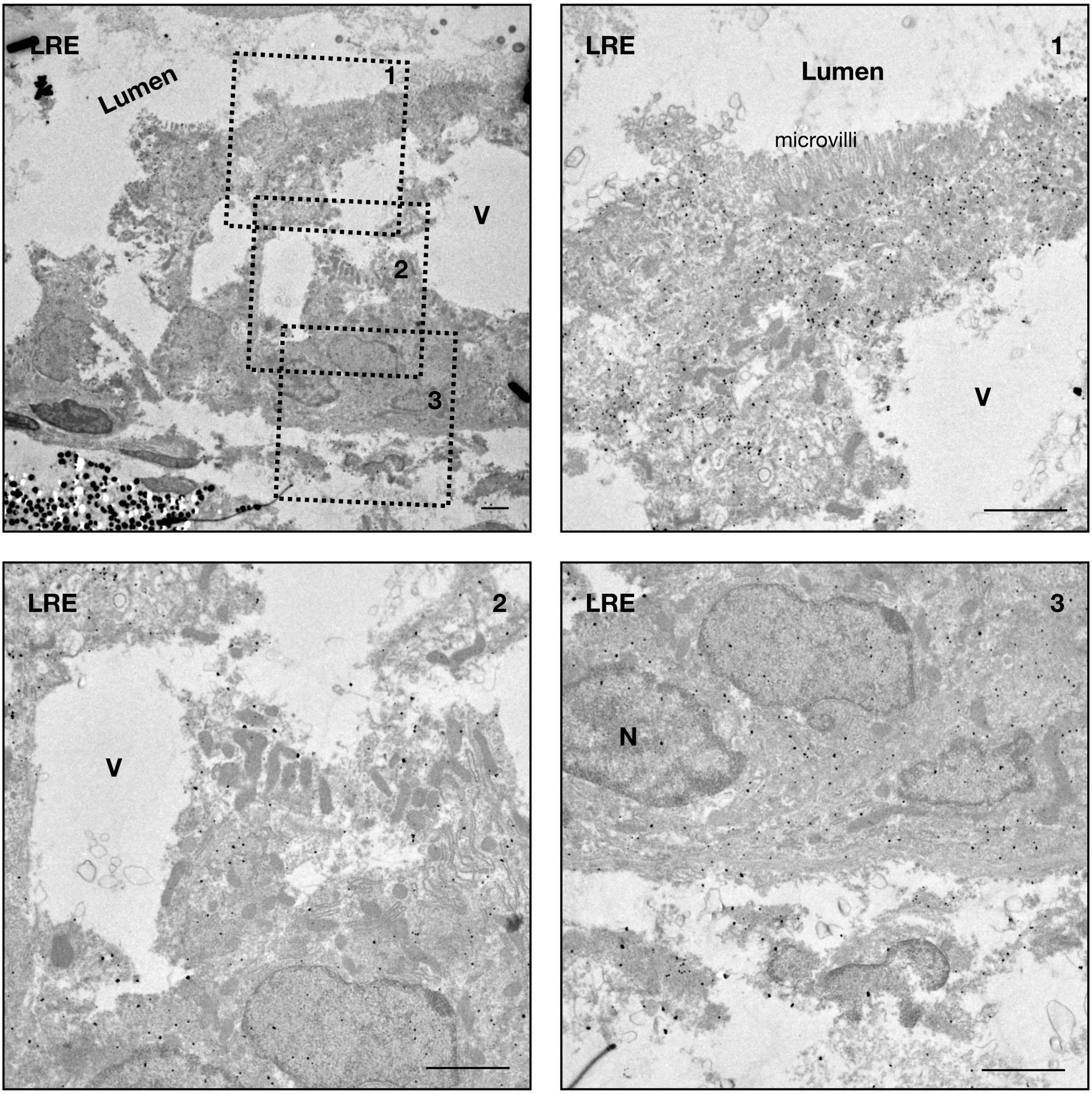

Supplementary Figure 2

Spatial distribution of Dr-Vsp within LREs.

Representative images of pre-embedding Dr-Vsp immunogold staining in LREs from 14-dpf wild-type zebrafish larva, demonstrating the spatial distribution of Dr-Vsp signals at multiple areas within the cell. Dr-Vsp is highly expressed at the subapical region (inset 1) and gradually decreased in the middle (inset 2) and basal part (inset 3) of LREs, corresponding to the immunostaining results in Figure 3. Note that non-specific signals were occasionally found scattered with low density inside and outside the tissue around the basal area (inset 3). Scale bar = 2 μm. V, vacuole. N, nucleus.

Supplementary Figure 3

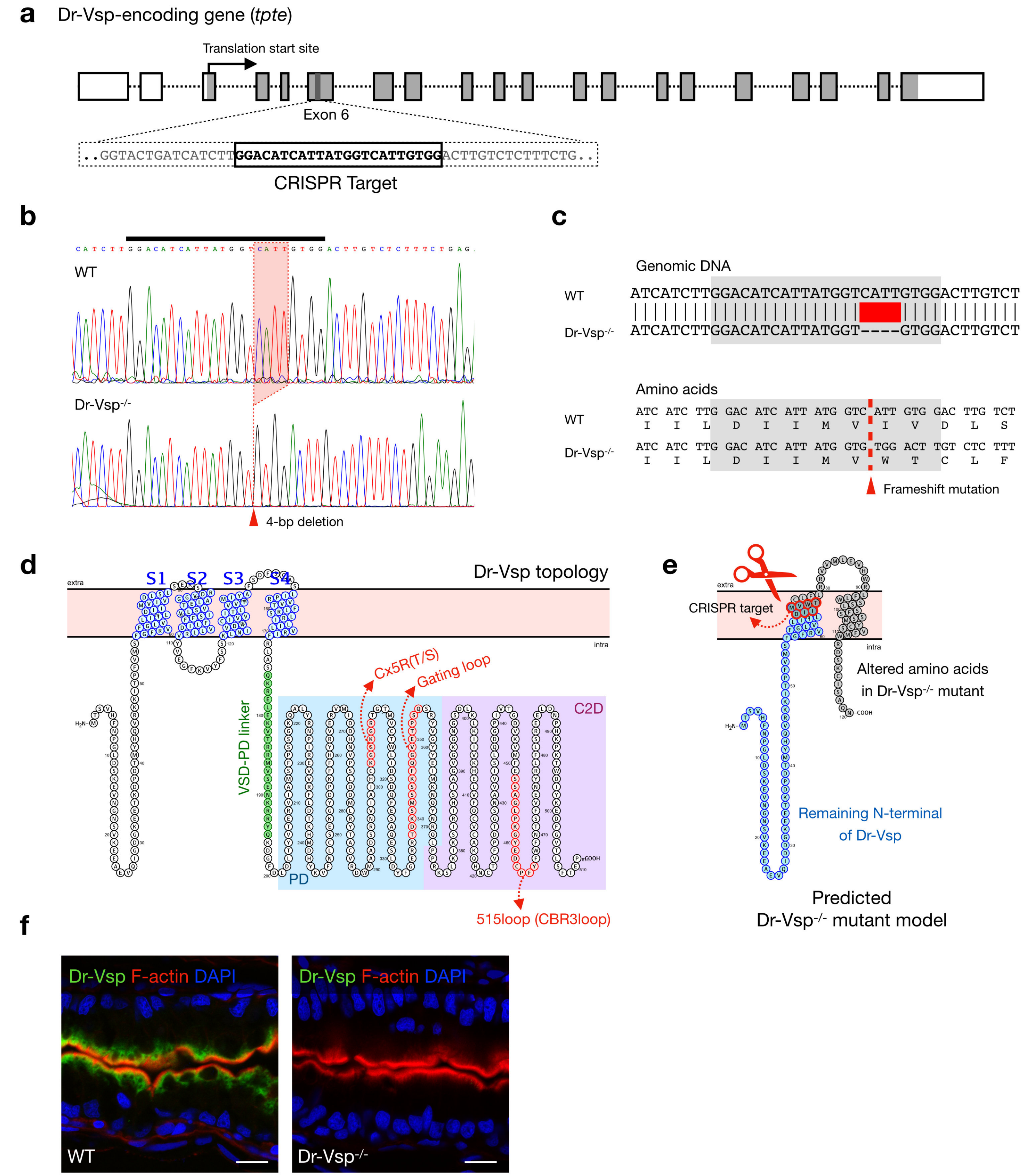

Supplementary Figure 3

Experimental schemes of CRISPR-Cas9-mediated Dr-Vsp$^{-/-}$ mutagenesis.

(a – e) Generation of heritable Dr-Vsp$^{-/-}$ zebrafish. (a) Schematic diagram of the zebrafish Dr-Vsp-encoding gene (*tpte*), consisting of 21 exons. The CRISPR target with the protospacer adjacent motif (PAM) sequence (TGG) is indicated in the box.

(b) Sequencing results illustrate microdeletion (arrowhead) in the genomic sequence of Dr-Vsp$^{-/-}$ zebrafish. WT, wild type.

(c) Alignment of genomic DNA sequences (top) and amino acid sequences (bottom) comparing between wild-type and Dr-Vsp$^{-/-}$ zebrafish. Microdeletion (red) within the CRISPR target (gray shade) induced frameshift mutation in the subsequent amino acids of Dr-Vsp$^{-/-}$ zebrafish.

(d) Dr-Vsp topology model illustrates the full-length 511 amino acids with the putative transmembrane helices (S1 - S4), Voltage sensor domain (VSD)-PD linker, and essential functional domains in the cytoplasmic catalytic region (Cx5R(T/S), gating loop, and 515 loop/CBR3 loop), based on the data published by Okamura et al[2]. Phosphatase domain (PD) is approximately indicated by a blue background, and C2 domain (C2D) by a purple background.

(e) Dr-Vsp$^{-/-}$ mutant model illustrates 120 amino acids remaining after frameshift mutation. The N-terminal region (prior to CRISPR target T1) contains 73 amino acids that are unchanged from the wild-type Dr-Vsp, whereas the subsequent 43 amino acids are altered and early terminated. Typical voltage-sensing residues (in S4) and the cytoplasmic catalytic region were missing.

(f) Immunostaining of wild-type (WT) and Dr-Vsp$^{-/-}$ zebrafish. Anti-Dr-Vsp antibody is specific to the S4 transmembrane helix and the cytoplasmic catalytic region. Dr-Vsp-postitive cell is undetectable in Dr-Vsp$^{-/-}$ enterocytes. Green, Dr-Vsp. Red, F-actin. Blue, DAPI. Scale bar = 10 µm

Supplementary Figure 4

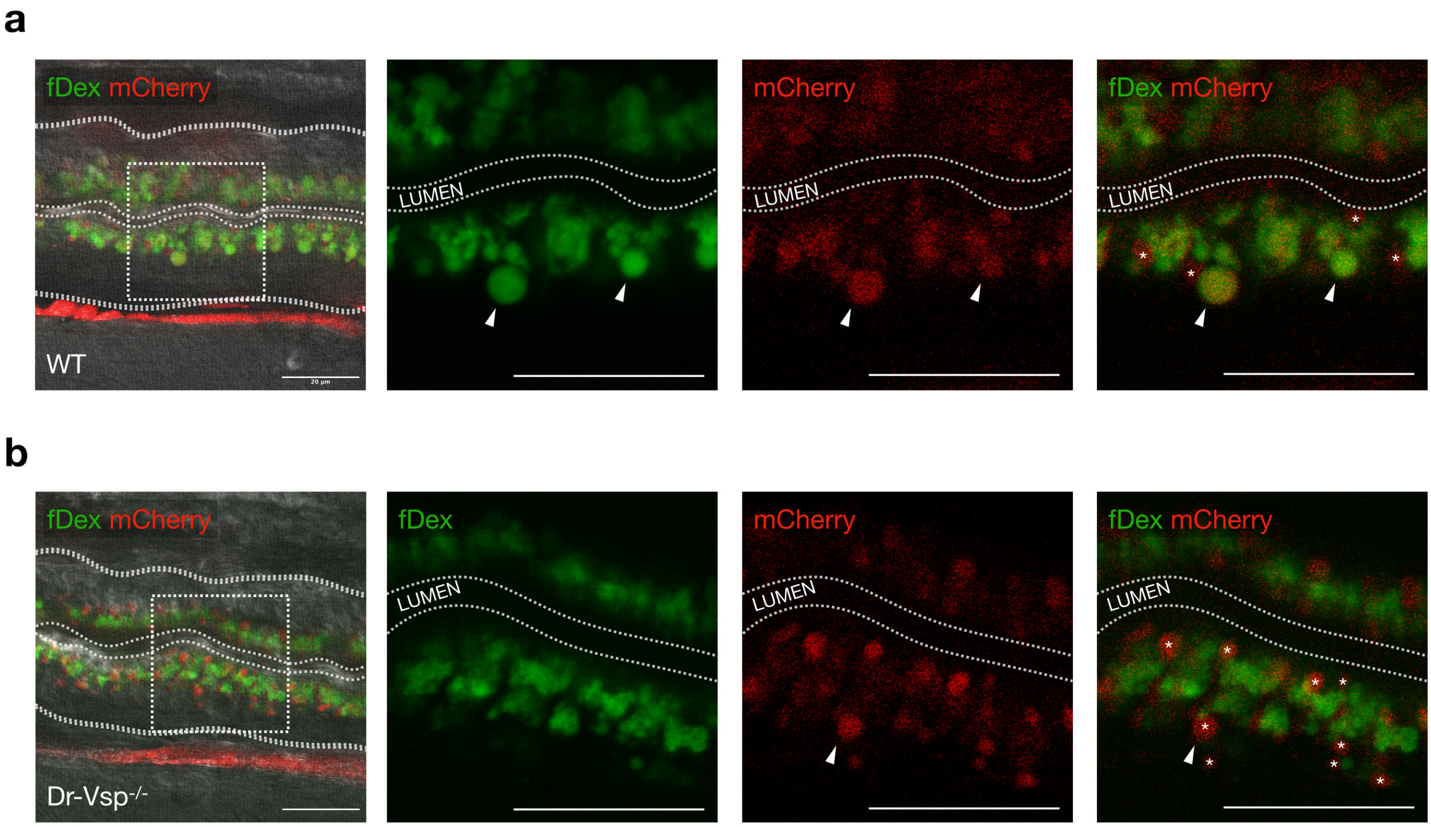

Supplementary Figure 4

Preliminary results of fDex-mCherry mixture internalization into zebrafish LREs after gavage.

(a) Live confocal images of wild-type (WT) LREs showing fDex-mCherry mixture internalization. Both fDex and mCherry signals are located in apical vesicles and supranuclear vacuoles (arrowheads). Some mCherry signals did not overlap with fDex signals (as indicated by asterisks).

(b) Live confocal images of Dr-Vsp$^{-/-}$ LREs showing fDex-mCherry mixture internalization. Notably, mCherry was transported deeply into some supranuclear vacuoles (arrowheads), whereas fDex was mostly found in apical vesicles. Dotted lines indicate the outlines of larval intestines. Many mCherry signals did not overlap with fDex signals (as indicated by asterisks). The results seem to suggest that mCherry and fDex were uptaken via different mechanisms into distinct intracellular vesicles, and the mismatch between mCherry and fDex signals was more pronounced in Dr-Vsp$^{-/-}$ than wild-type LREs. Scale bar = 20 µm.

Supplementary Figure 5

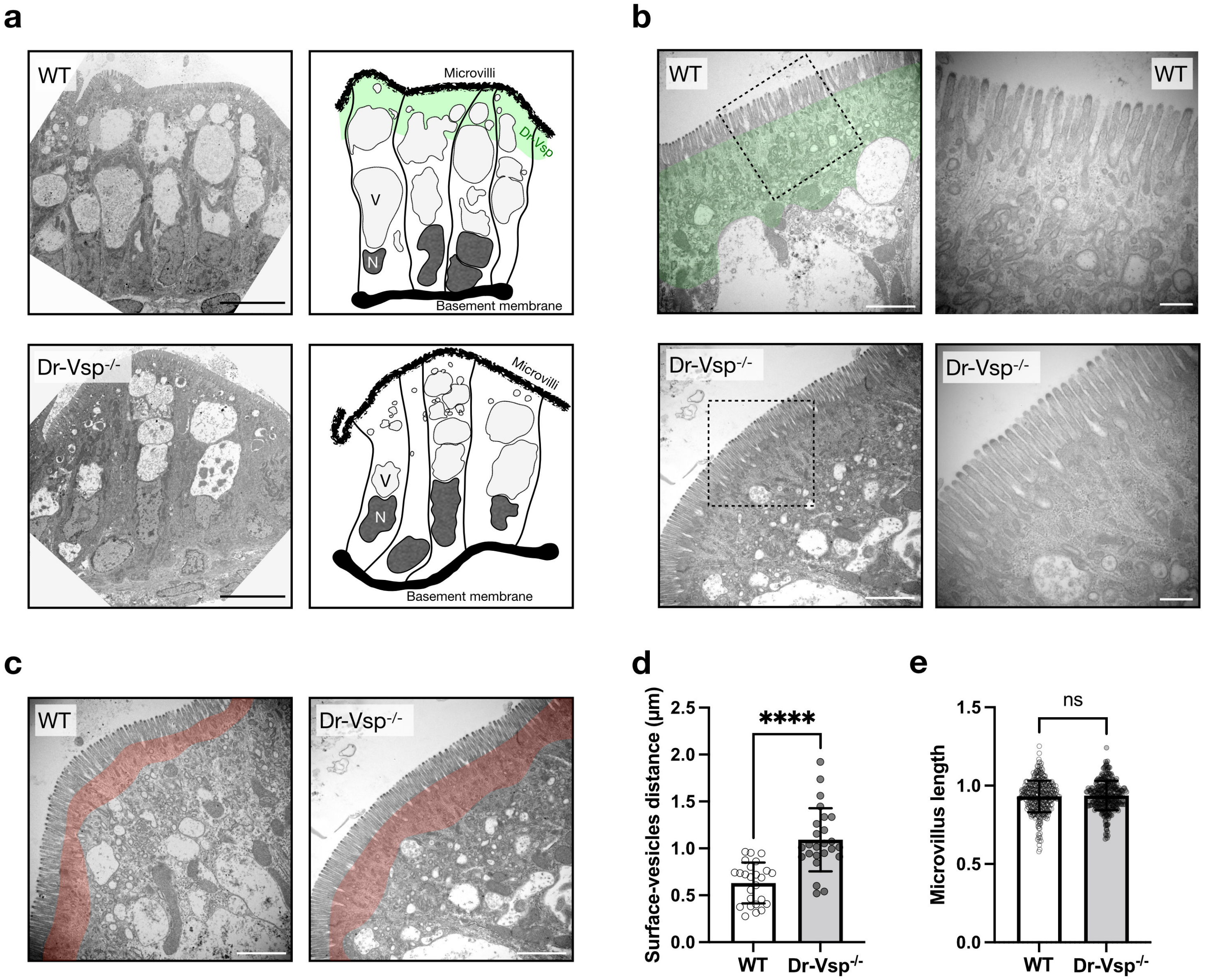

Supplementary Figure 5

Morphological structures of zebrafish LREs under transmission electron microscopy.

(a) Representative TEM images (left) of LREs from 14-dpf wild-type (top) and Dr-Vsp$^{-/-}$ (bottom) zebrafish larvae; and their corresponding schematic illustration (right). Green color represents the subapical region upper to supranuclear vacuoles, where the positive immunofluorescence signal of Dr-Vsp was observed in Figure 3, a and b. N, nucleus. V, vacuole. Scale bar = 10 μm.

(b) Representative TEM images of LREs from 14-dpf wild-type (top) and Dr-Vsp$^{-/-}$ (bottom) zebrafish larvae, showing the ultrastructures of microvilli and subapical region. Key features of absorptive enterocytes are presented, including membrane invaginations at inter-microvillous spaces, cytoplasmic tubules and tubule-vacuole complexes, and numerous endocytic vesicles. In wild-type LRE, green color represents the subapical region upper to supranuclear vacuole, where immunofluorescence signal of Dr-Vsp was observed in Figure 3, a and b. Scale bar = 2 μm (left) and 500 nm (right).

(c) Representative TEM images of LREs from 14-dpf wild-type (left) and Dr-Vsp$^{-/-}$ (right) zebrafish larvae, showing the ultrastructures of microvilli and subapical region. Red color represents the area beneath apical surface that contains cytoplasmic tubules but few well-defined endocytic vesicles. The vesicles are more densely distributed in the areas deeper than the red region and are frequently associated with larger endosomal vacuoles.

(d) Inter-edge distance between apical surface and vesicle-dense area in wild-type and Dr-Vsp$^{-/-}$ LREs, corresponding to the width of red shading areas shown in (c). Enterocytes, > 50 cells from 25 TEM images (magnification = 15000x) for each zebrafish line. Data were collected from 4 wild-types and 3 Dr-Vsp$^{-/-}$ larvae. Average distances in each TEM image were analyzed using ImageJ macro.

(e) Microvillous length of wild-type and Dr-Vsp$^{-/-}$ LREs measuring at TEM level from the same samples as in (d). Error bars, means ± SD; ****P < 0.0001; unpaired Student's t-test; ns, no statistically significant difference.

Supplementary Figure 6

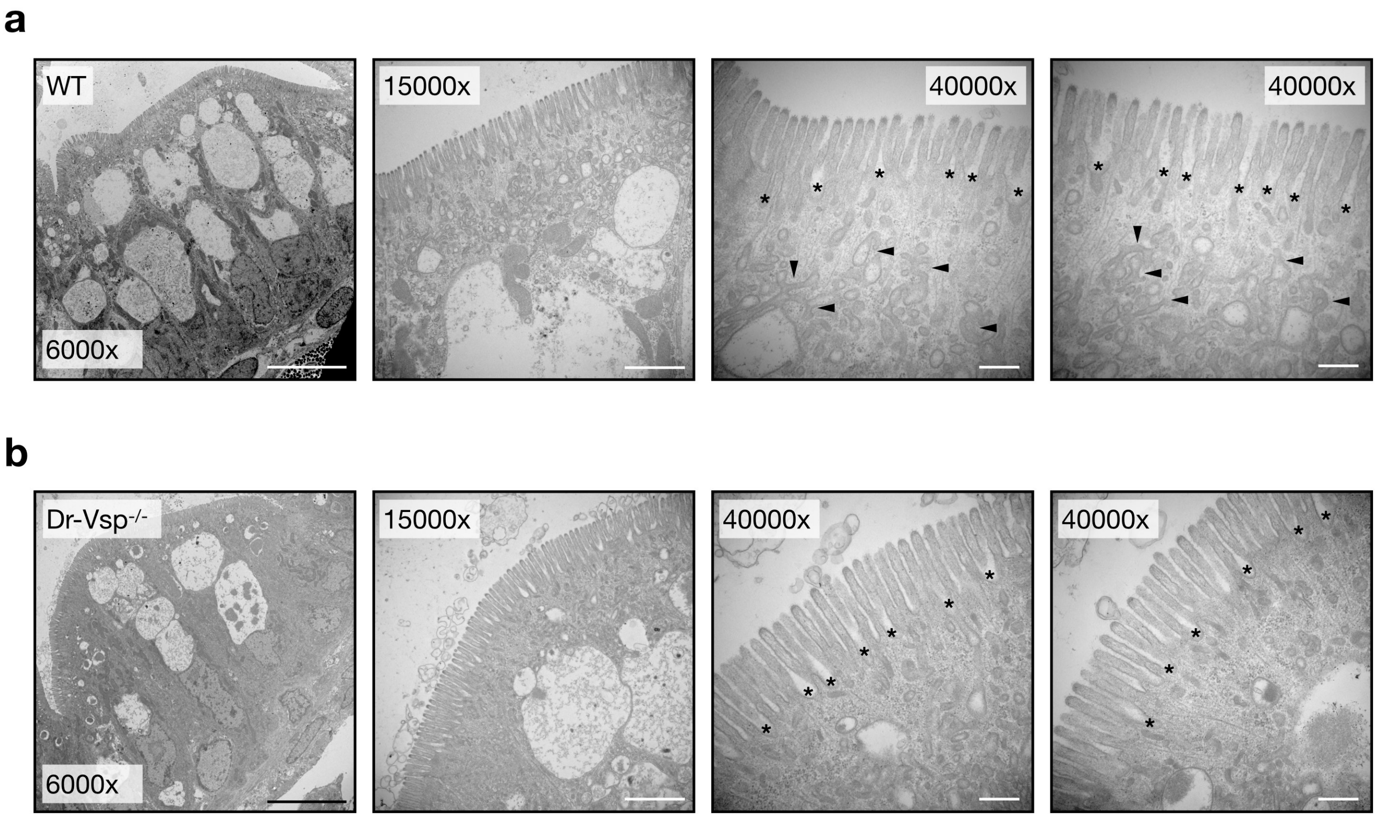

Supplementary Figure 6

TEM image series of wild-type and Dr-Vsp$^{-/-}$ LREs.

Representative TEM image series of LREs from (a) 14-dpf wild-type; and (b) Dr-Vsp$^{-/-}$ zebrafish larvae. Large vacuoles are distributed in the supranuclear region but are fewer in Dr-Vsp$^{-/-}$ LREs. Membrane invaginations at inter-microvillous spaces (*) can be observed at higher magnification. Branching cytoplasmic tubules and tubule-vacuole complexes (arrowheads) are more common in wild-type LREs. Scale bar at 6000x = 10 µm; at 15000x = 2 µm; at 40000x = 500 nm.

Supplementary Figure 7

```
Hs-Vsp1         --------------------------------------------------------------------------------
Mm-Vsp     1    MYGEKKSHLYLWMEHYGYDMPANIYKMYSQPSRKTDDANKKVSVSASRTIKLNGSTGYDTNEQITLITNGSSLSYPDEIK  80
Dr-Vsp          --------------------------------------------------------------------------------
Cc-Vsp          --------------------------------------------------------------------------------
On-Vsp          --------------------------------------------------------------------------------
Ol-Vsp          --------------------------------------------------------------------------------
Ci-Vsp     1    M--------------EGFD--------------------------GSDFSPPADLVGVDG---------AVMRNVVD---  28
Ap-Vsp     1    MC-------------QGIC-----------------------EIMADDTSPHRYLIDAEEENEPTLPLPTPLQDVPS---  41

Hs-Vsp1    1    ----------------------------------------------------MNESPQTNEFK-GTTEEAPAKESPHT--  25
Mm-Vsp     81   SASYADPISTKAYTNDSSVYDPGGASSSTTLYELNSLSEVSKEIITQGESALLRDKEATSELKIPSTLQTQTSMSTNTLS  160
Dr-Vsp     1    ------------------------------------------------------------------------------MT  2
Cc-Vsp     1    ------------------------------------------------------------------------------MT  2
On-Vsp     1    ------------------------------------------------------------------------------MS  2
Ol-Vsp          ------------------------------------------------------------------------------M-
Ci-Vsp     29   -----------VTINGDVTAPPKAAPRK-----SESVKKVHWNDVDQG----PSEKPETRQEE---RI------------  73
Ap-Vsp     42   -----------TNVLAHSIFPPPVEPESKSDTSSGECKAVHL-VMTNGFLS------ANAEIGLDDTVPLGGRL------  97

                                                                          S1
Hs-Vsp1    26   -SEFKGAALVSPISKSMLE-RLSKFEVED--AEN--VASYDSKIKKIVHSIVSSFAFGIFGVFLVLLDVTLLLADLIFTD  99
Mm-Vsp     161  LSDLSSDYQEEQMNKCKLN-QMSKLYDDD--ERTDIQKSYWNVVKKFVRILVSSVAFRIFGIFLVILDVFLVVVDLNVSE  237
Dr-Vsp     3    SVHFNPGLDSKEVNGNSVK-EEAEVQIDDGKEETKDPDTMYHQVRKKITPFVMSFGFRVFGLVLIILDIIMVIVDLSLSE  81
Cc-Vsp     3    SVHFNPGLDSKEVNGNGLK-EEAEVQIDDGREDTEDPDTMYHRVRKTIAPFVMSFGFRVFGLVLIIVDIILVIVDFSLSN  81
On-Vsp     3    SVYFNPGSDSS-VNGNITKMENAKVEIDDGKEESALPDTLYYNLRKKIAPFVMSFGFRLFGVILIIVDFVLVIVDLSLPA  81
Ol-Vsp     2    SVHFQPGSDAG-VNGNVAKMEDAKVEIDNGKEESALPDTLYHNIQRAITPFVMSFGFRLFGVVLIIVDFVLVIVDFSLAS  80
Ci-Vsp     74   --------DIPEISGLWWGEN--EHGVDDGRME--IPTTGVGRVQFRVRAVIDHLGMRVFGVFLIFLDIILMIIDLSLPG  141
Ap-Vsp     98   -LHDDPGENDEE---HWTENGHVQFELGDRAHVSVIRYPRLKKARMRTKRVVEHLAVRLIGVGLVIFDIIIVILDIVFNP  173

                          S2                                S3                     S4 →
Hs-Vsp1    100  SKLYIPLEYRSISLAIGLFFLMDVLLRVFVEGRQQYFSDLFNILDTAIIVIPLLVDVIYIFFDI----KLLRNIPRWTHL  175
Mm-Vsp     238  KKIYIPLDYRSISLAIALFFLVDVLLRVSVEGRRRYFSDVLNTLDAVVIGVTVVVAVIYALYDK----HFLRDIPRLAVL  313
Dr-Vsp     82   KSRDVGGAPETVSLVISFFFLIDVLLRVYVEGFKVYFSSKLNIVDACIVVITLVVTMIYAFSDF----SGASLIPRVVTF  157
Cc-Vsp     82   GSHDVRRAMESVSLVISFFFLIDVLLRVYVEGFKVYFSSKLNIMDACIVVVTLVVTMIYAFTDL----SGASLIPRVVTF  157
On-Vsp     82   KSRDAGNALEAVSLTISFFFLADVLLRVYVEGFKVYFSSKLNIIDACVVIVTLAVTMVYTFSDL----SGTSLIPRVVTF  157
Ol-Vsp     81   KSREVAKALEAVSLSISFFFLADVLLRVYVEGFKVYFRSKLNIVDACVVVLTLVVTMVYTFSDL----SGVSLIPRVVNF  156
Ci-Vsp     142  KSESSQSFYDGMALALSCYFMLDLGLRIFAYGPKNFFTNPWEVADGLIIVVTFVVTIFYTVLDEYVQETGADGLGRLVVL  221
Ap-Vsp     174  QNIATMEAYDAISVAVWCYFLIEICLRIFAKG-KKFFRTKLDLLDLLIVTVTGSVTIVYVSVDL------TGSYLKLVVV  246

                →S4                  VSD-PD linker
Hs-Vsp1    176  VRLLRLIILIRIFHLLHQKRQLEKLMRRLVSENKRRYTRDGFDLDLTYVTERIIAMSFPSSGRQSFYRNPIEEVVRFLDK  255
Mm-Vsp     314  LRPLRLLILIRILQLAHQKRQLERLTRKLVSGNKRRYKKDGFDLDLTYVTERIIAMSFPSSGRESFYRNPIKEVVRFLDT  393
Dr-Vsp     158  LRSLRILILVRIFRLASQKRELEKVTRRMVSENKRRYQKDGFDLDLTYVTERVIAMSFPSSGKQALYRNPIREVVRFLDT  237
Cc-Vsp     158  LRSLRILILVRVFRLASQKKELEKVTRRMVSENKRRYQKDGFDLDLTYVTDRVIAMSFPSSGKQALYRNPIREVARFLDT  237
On-Vsp     158  LRFLRIIILVRVFRLAAQKKELEKVTRRMISENKRRYQKDGFDLDLTYVTDRVIAMSFPSSGKQAFYRNPIREVARFLDT  237
Ol-Vsp     157  FRFLRIIILVRVFRLAAQKKELEKVTRRMISENKRRYQKDGFDLDLTYVTDRVIAMSFPSSGKQAFYRNPIKEVARFLDT  236
Ci-Vsp     222  ARLLRVVRLARIF---YSHQQMKASSRRTISQNKRRYRKDGFDLDLTYVTDHVIAMSFPSSGRQSLFRNPIGEVSRFFKT  298
Ap-Vsp     247  FRVLRIFLLVRLL---SERKHVSKATRKMVSQNKRRYREDDFDLDLTYITDRVIASSFPSSGSQAIYRNPIEEVARFLDT  323

                                                                          Cx5R(T/S)
Hs-Vsp1    256  KHRNHYRVYNLCSERAYDPKHFHNRVSRIMIDDHNVPTLHEMVVFTKEVNEWMAQDLENIVAIHCKGGKGRTGTMVCALL  335
Mm-Vsp     394  KHPNHYQVYNLCSERAYDPKHFHYRVRRIMIDDHNVPTLEEMLLFSKEVNNWMAQDPENVVAIHCKGGKGRTGTMVCACL  473
Dr-Vsp     238  KHMDHYKVFNLCSEKGYDPKFFHYRVERVMIDDHNVPSLDDMLRYTACVRDWMAADSRNVIAIHCKGGKGRTGTMVCTWL  317
Cc-Vsp     238  KHLDHYKVFNLCSEKGYDPKFFHYRVERVMIDDHNVPSLEDMLRYTACVRDWMAADSSNVIAIHCKGGKGRTGTMVCTWL  317
On-Vsp     238  KHEGHYKVYNLCSEKGYDPQFFHYRVERVFIDDHNVPSLEDMLKYTASVREWMSADPQNIIAIHCKGGKGRTGTMVCTWL  317
Ol-Vsp     237  KHEGHYKVFNLCSEKGYDPQHFHYRVERVFIDDHNVPALEDMLKFTASVREWMSADPQNVIAIHCKGGKGRTGTMVCTWL  316
Ci-Vsp     299  KHPDKFRIYNLCSERGYDETKFDNHVYRVMIDDHNVPTLVDLLKFIDDAKVWMTSDPDHVIAIHCKGGKGRTGTLVSSWL  378
Ap-Vsp     324  KHPDHYKVYNLCIERHYDESFFHNRVGRVLIYDHNVPRLKDLVAFCADARQWMEADNKNILFIHCKGGKGRTGLAVCAWL  403

                                   Gating loop
Hs-Vsp1    336  IASEIFLTAEESLYYFGERRTNKTHSNKFQGVETPSQNRYVGYFAQVKHLYNWNLPPRRILFIKRFIIYSI----RGDVC  411
Mm-Vsp     474  IASEIVLNAKESLYFFGERRTDKSNSSKFQGIETPSQNRYVKYFEKLKINYQLTLPPKKVLVIKRLVVYSIHGVGKGDGS  553
Dr-Vsp     318  IDSDQFESAQESLDYFGERRTDKSMSSKFQGVETPSQSRYVGYYEIMKNQYNRQLPPRKSLKIKSIRIHSIAGVGKGNGS  397
Cc-Vsp     318  IDSDQFESAQESLDYFGERRTDKSMSSKFQGVETPSQSRYVGYYEIMKNQYNRQLPPQKSLKIKSIRIHSIAGVGKGNGS  397
On-Vsp     318  IDSDQFESAQDSLEYFGERRTDKSQSSKFQGVETPSQSRYVGYYEIMKNKLNRQLPPPKSLRIKSIRIHSIAGVGKGNGS  397
Ol-Vsp     317  IDSDQFESAQDSLEYFGERRTDKTRSSKFQGVETPSQSRYVGYYEVMKNKFNRQLPPPQSLRIKSLRIHSIAGVGRGDGS  396
Ci-Vsp     379  LEDGKFDTAKEALEYFGSRRTDFEVGDVFQGVETASQIRYVGYFEKIKKNYGGQLPPMKKLKVTGVTITAIQGVGRGNGS  458
Ap-Vsp     404  LESGVFTDTKECMGYFGVRRTDYTEGDMYQGVDTPSQSRYVDYFQKIKYSLGGHVPSPTPLTINTVRISGIQGVGKGDGR  483

                                                                      515loop (CBR3loop)
Hs-Vsp1    412  DLKVQVVMEKKVVFSS--TSLGNCSILHDIETDKILINVYDGPPLYDDVKVQFRSS--NLPKYYDNCPFFFWFNTSFIQN  487
Mm-Vsp     554  DLEVQIIMWQETVFSF--CNSRNCMIFHDPETDRAIINVFHCPALYDDVKVKFLSP--NLPKYYDDCPFFFWFHTSFIKN  629
Dr-Vsp     398  DLKLKIIVKHELVFQCVCAKQHNCTVFPDTGSNAVVISLQDGPIVTGDVKVMFRSSA-GLPKGYEDCPFYFWFNTSFVEN  476
Cc-Vsp     398  DLKVKIIVKRELVFQCVCAKQQNCAVFPDTGNNAVVISLQEGPVVTGDVKVMFRSSA-GLPKGYEDCPFYFWFNTSFVEN  476
On-Vsp     398  DFKVKIVVRKELVFECVCAKQENCTVFPDVGNNAAVISLQNGPVVEGDVKVMFRSSA-GLPKGYEDVPFYFWFNTSFIED  476
Ol-Vsp     397  DLKVKIIVRGELVFQCVCAQQENCTVFPDAGSNAAVISLQNGPVVEGDVKVMFRSSA-GLPKGYEDVPFYFWFNTSFVEH  475
Ci-Vsp     459  DLSMQIVSERQEVLLCKFAEGYNCALQYDATDDCVTCEVKNCPVLAGDIKVRFMSTSKSLPRGYDNCPFYFWFNTSLVEG  538
Ap-Vsp     484  DLWMEVHSDEVSLFSCDFRCNINCSVVYDFDMDTVIIQVKNSPAFSGDVQIKFKSSNKKIPKAYGECAFFFWFYTGFISD  563

Hs-Vsp1    488  NRLCLPRNELDNPHKQKAWKIYPPEFAVEILFGEK-----  522
Mm-Vsp     630  NRLYLPRNELDNTHKPKTWKIYGEKFAVEVDFGEN-----  664
Dr-Vsp     477  YRLFLSREELDNPHKPKTWDIYKEDFGVTLSFTEP-----  511
Cc-Vsp     477  NSLYLSREELDNPHKSKTWDIYKEDFGVTLYFTDP-----  511
On-Vsp     477  NKLFLPREELDNPHKPKTWDLYKEDFGVTMYFSESE----  512
Ol-Vsp     476  NKLFFPREELDNPHKPKTWNLYKEDFGITVYFLPV-----  510
Ci-Vsp     539  DHVTLKREEIDNPHKKKTWKIYRDNFTVKLTFSDAEDI--  576
Ap-Vsp     564  NRLYLLRKDLDNPHKAEAWKVYRDTFAVDVTFGPVAESET  603
```

Supplementary Figure 7

Multiple amino acid alignment of full-length Vsp orthologs from human (*Homo sapiens*, Hs-Vsp1 or TPIP), mouse (*Mus musculus*, Mm-Vsp), zebrafish (*Danio rerio*, Dr-Vsp), common carp (*Cyprinus carpio*, Cc-Vsp), Nile tilapia (*Oreochromis niloticus*, On-Vsp), Japanese medaka (*Oryzias latipes*, Ol-Vsp), sea squirt (*Ciona intestinalis* Type A, Ci-Vsp), and Crown-of-thorns starfish (*Acanthaster planci*, Ap-Vsp). Boxes indicate the putative transmembrane helices (S1 - S4), Voltage sensor domain (VSD)-PD linker, CX5R(T/S), gating loop, and 515 loop/CBR3 loop[2]. Phosphatase domain (PD) is approximately indicated by blue letters, and C2 domain (C2D) by purple letters. Amino acid sequences were retrieved from National Center for Biotechnology Information (NCBI) public database. Analyses were performed using online Constraint-based Multiple Alignment Tool (COBALT) (https://www.ncbi.nlm.nih.gov/tools/cobalt/)

Supplementary Figure 8

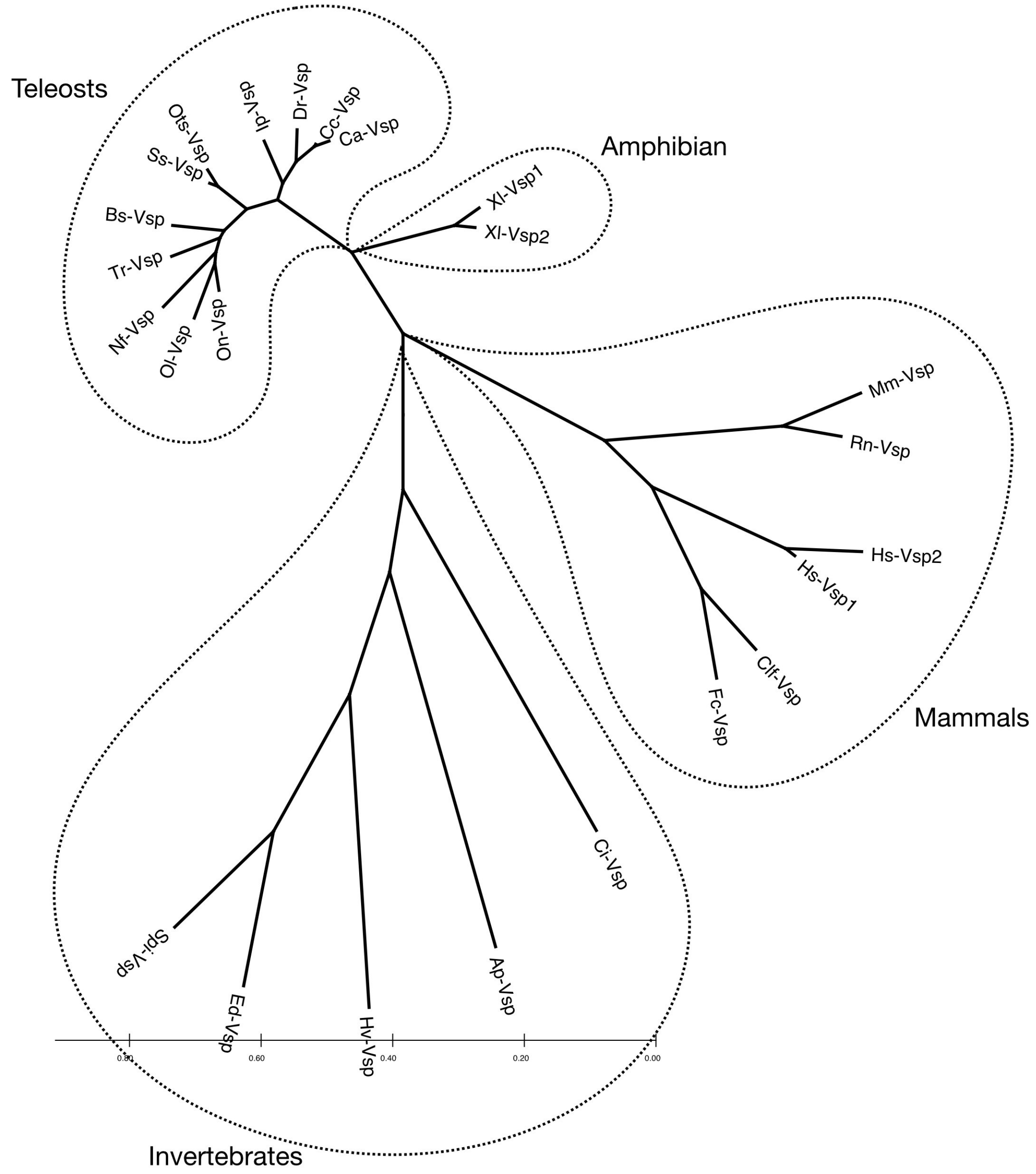

Supplementary Figure 8:

Molecular phylogenetic tree showing the phylogenic relationships among Vsp orthologs from **mammals:** human (*Homo sapiens*, Hs-Vsp1 or TPIP, and Hs-Vsp2 or TPTE), mouse (*Mus musculus*, Mm-Vsp), rat (*Rattus norvegicus*, Rn-Vsp), dog (*Canis lupus familiaris*, Clf-Vsp), cat (*Felis catus*, Fc-Vsp); **amphibian:** American clawed frog (*Xenopus laevis*, Xl-Vsp1 and Xl-Vsp2); **teleosts:** goldfish (*Carassius auratus*, Ca-Vsp), common carp (*Cyprinus carpio*, Cc-Vsp), zebrafish (*Danio rerio*, Dr-Vsp), channel catfish (*Ictalurus punctatus*, Ip-Vsp), Chinook salmon (*Oncorhynchus tshawytscha*, Ots-Vsp), Atlantic salmon (*Salmo salar*, Ss-Vsp), Siamese fighting fish (*Betta splendens*, Bs-Vsp), Japanese puffer (*Takifugu rubripes*, Tr-Vsp), turquoise killifish (*Nothobranchius furzeri*, Nf-Vsp), Japanese medaka (*Oryzias latipes*, Ol-Vsp), Nile tilapia (*Oreochromis niloticus*, On-Vsp); **invertebrates:** sea squirt (*Ciona intestinalis* Type A, Ci-Vsp), crown-of-thorns starfish (*Acanthaster planci*, Ap-Vsp), fresh-water polyp (*Hydra vulgaris*, Hv-Vsp), sea anemone (*Exaiptasia diaphana*, Ed-Vsp), and smooth cauliflower coral (*Stylophora pistillata*, Spi-Vsp). Data were retrieved from the same source as Supplementary Figure 7, and analyzed using Molecular Evolutionary Genetics Analysis (MEGA) X Software (Pennsylvania State University, Pennsylvania, US).

Supplementary Figure 9

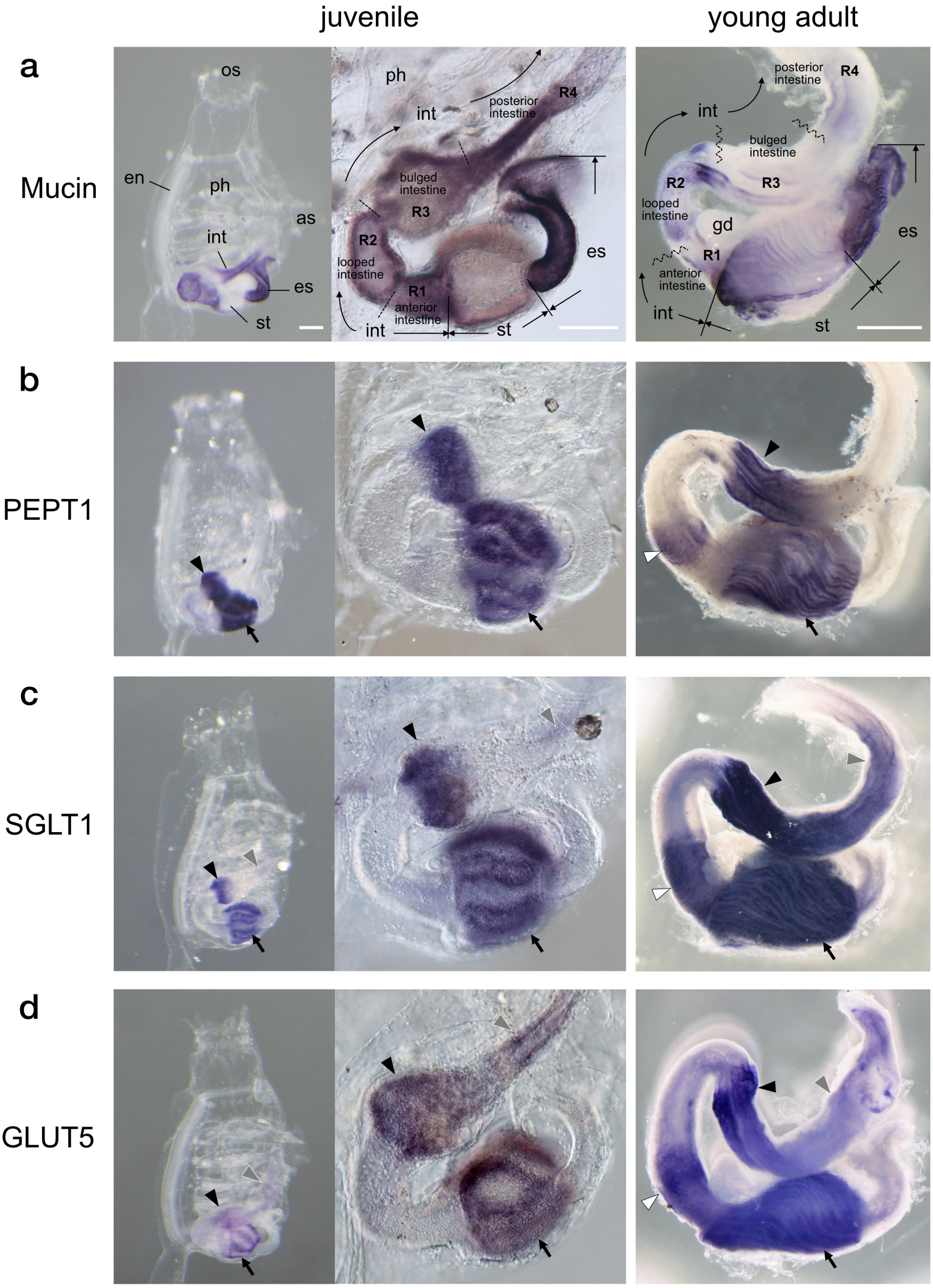

Supplementary Figure 9

Morphological structure of ascidian intestine and expression profiles of intestine-related genes

(a – d) Expression profiles of intestine-related genes in *Ciona* juvenile (1st column) and young adult (2nd column) revealed by WISH. In the juvenile, whole (left panel) and magnificent of the post-pharyngeal region of the digestive tract including esophagus, stomach, and intestine (right panel) were shown.

(a) Morphological structure of the digestive tract was noted on the expressions of *Mucin* gene. Digestive tract of *C. intestinalis* Type A was divided into oral siphon (os), pharynx (ph), esophagus (es), stomach (st), and intestine (int)[3]. The intestine was subdivided into four regions, R1 to R4[4]. *Mucin* gene was expressed broadly in the post-pharyngeal region of the digestive tract of the juvenile, but not expressed in the stomach and bulged-intestine (R3). In the young adult specimen, expression profile was similar to the juvenile, but expression signal was disappeared between anterior intestine (R1) and looped-intestine (R2) beside the developing gonad (gd).

(b) Absorption-related *PEPT1* gene was invested as an example of the peptide transporter genes. Expression signals of the *PEPT1* were observed in stomach (arrow) and bulged-intestine (R3) of the mid-intestine (arrowhead) in both developmental stages of juvenile and young adult. Additional expression (white arrowhead) was appeared between anterior intestine (R1) and looped-intestine (R2) of the young adult intestine.

(c) Absorption-related *SGLT1* gene was invested as a secondary active glucose transporter. Transcripts of the *SGLT1* were detected in stomach (arrow), bulged-intestine (R3) (arrowhead), and posterior-intestine (R4) (gray arrowhead) in juvenile and young adult specimens.

(d) *GLUT5* gene was also assessed as a facilitated transporter of the monosaccharide. Transcripts of the *GLUT5* were detected in stomach (arrow), bulged-intestine (R3) (arrowhead), and posterior-intestine (R4) (gray arrowhead) in juvenile and young adult specimens. Additional expression (white arrowhead) between anterior intestine (R1) and looped-intestine (R2) was also found in the young adult. Scale bars = 200 μm in (left) and 1 mm in (right). Additional abbreviations: as, atrial siphon; en, endostyle. Latest gene model IDs (HT version) of the *Ciona Mucin*, *PEPT1*, *SGLT1*, and *GLUT5* are KY.Chr2.2226, KY.Chr13.370, KY.Chr10.1303, and KY.Chr7.810, respectively[5]

Supplementary Table 1

RT-PCR Primers used in this study

Target gene	Primer (5' – 3')	
tpte	Sense	CAGAGCAGGTATGTTGGCTA
	Antisense	CAGCACTGGACTCAAACATGA
β-actin	Sense	GGTATGGAATCTTGCGGTAT
	Antisense	GGTATGGAATCTTGCGGTAT

Supplementary Table 2

CRISPR target sequences of *tpte* and PCR primers used in this study

Target sequence		Primer (5' – 3')	
T1	GGACATCATTATGGTCATTG**TGG**	Sense	GGTGAGTGGCATACTGGAATTT
		Antisense	TCCACATAAACACGGAGCAATA
T2	GAGAAGAGTCGTGATGTTGG**AGG**	Sense	TTATGTTTGCAGTGTTTTTGGC
		Antisense	ACATCATGTGCTGAAATCAAGG
T3	TGCCACTCACCGAAAACCAA**AGG**	Sense	TCTGATTTGTTTTGCAGTCAGG
		Antisense	CCAACATCACGACTCTTCTCAG

Bold letters at the end of each target sequence indicate the protospacer adjacent motif (PAM) sequence.

Supplementary Table 3: Key resources table

REAGENT or RESOURCE	SOURCE	IDENTIFIER
**Antibodies**		
Mouse monoclonal Anti-Zebrafish VSP/TPTE (clone N432/21)	UC Davis/NIH NeuroMab Facility	Cat# 73-485; RRID: AB_2716253
Rabbit monoclonal Anti-Sodium Potassium ATPase	Abcam	Cat# ab76020; RRID: AB_1310695
Acti-stain™ 488 Phalloidin	Cytoskeleton, Inc.	Cat# PHDG1
Alexa Fluor™ 594 Phalloidin	Invitrogen	Cat# A12381; RRID: AB_2315633
**Chemicals, peptides, and recombinant proteins**		
Dextran, Alexa Fluor™ 488-tagged; 10,000MW	Molecular Probes, Inc.	Cat# D22910
mCherry solution	This study	N/A
TRIzol™ LS reagent	Invitrogen	Cat# 10296010
Anti-Digoxigenin-AP, Fab fragments	Roche	Cat# 11093274910
BCIP/NBT solution	Wako	Cat# 022-16231
BCIP/NBT solution	Roche	Cat# 11681451001
Agarose, Ultra-low Gelling Temperature	Sigma-Aldrich	Cat# 9012-36-6
**Critical commercial assays**		
SuperScript™ III First-Strand Synthesis System	Invitrogen	Cat# 18080051
PCR DIG Probe Synthesis Kit	Roche	Cat# 11636090910
BigDye™ Terminator v3.1 Cycle Sequencing Kit	Thermo Fisher	Cat# 4337457
Lipofectamine™ 3000 transfection reagent	Invitrogen	Cat# L3000015

**Experimental models: Cell lines**		
MDCK-II Cell Line canine	ATCC	CRL-2936
**Experimental models: Organisms/strains**		
Zebrafish/ RIKEN wild-type	RIKEN Brain Science Institute	N/A
Zebrafish/ *Tg(tpte:EGFP)*	This study	N/A
Zebrafish/ Dr-Vsp$^{-/-}$	This study	N/A
Ciona intestinalis Type A	This study	N/A
**Oligonucleotides**		
RT-PCR Primers used in this study	This study; Supplementary Table 1	N/A
CRISPR target sequences of *tpte* and PCR primers used in this study	This study; Supplementary Table 2	N/A
**Recombinant DNA**		
Mbait-hsp70 promoter-EGFP-polyA	Kimura et al.[1]	N/A
mCherry-Dr-VSP	This study	N/A
EGFP-Rab5	This study	N/A
EGFP-Rab11	This study	N/A
**Software and algorithms**		
ImageJ	NIH Image	http://imagej.nih.gov/ij

GraphPad Prism 8	GraphPad Software	https://www.graphpad.com
MEGAX	MEGA	https://www.megasoftware.net
COBALT	NIH NCBI	https://www.ncbi.nlm.nih.gov/tools/cobalt
iDEP v0.92	Ge et al.[6]	http://bioinformatics.sdstate.edu/idep92

Supplementary References

1 Kimura, Y., Hisano, Y., Kawahara, A. & Higashijima, S. Efficient generation of knock-in transgenic zebrafish carrying reporter/driver genes by CRISPR/Cas9-mediated genome engineering. *Sci Rep* **4**, 6545, doi:10.1038/srep06545 (2014).

2 Okamura, Y., Kawanabe, A. & Kawai, T. Voltage-Sensing Phosphatases: Biophysics, Physiology, and Molecular Engineering. *Physiol Rev* **98**, 2097-2131, doi:10.1152/physrev.00056.2017 (2018).

3 Chiba, S., Sasaki, A., Nakayama, A., Takamura, K. & Satoh, N. Development of *Ciona intestinalis* juveniles (through 2nd ascidian stage). *Zoolog Sci* **21**, 285-298, doi:10.2108/zsj.21.285 (2004).

4 Yoshida, R. & Sasakura, Y. Establishment of enhancer detection lines expressing GFP in the gut of the ascidian *Ciona intestinalis*. *Zoolog Sci* **29**, 11-20, doi:10.2108/zsj.29.11 (2012).

5 Satou, Y. *et al.* A nearly complete genome of *Ciona intestinalis* Type A (*C. robusta*) reveals the contribution of inversion to chromosomal evolution in the genus *Ciona*. *Genome Biol Evol* **11**, 3144-3157, doi:10.1093/gbe/evz228 (2019).

6 Ge, S. X., Son, E. W. & Yao, R. iDEP: an integrated web application for differential expression and pathway analysis of RNA-Seq data. *BMC Bioinformatics* **19**, 534, doi:10.1186/s12859-018-2486-6 (2018).
