# Supplementary material for: Voltage-sensing phosphatase (Vsp) regulates endocytosis-dependent nutrient absorption in chordate enterocytes
Source: Commun Biol. 2022 Sep 10;5:948. doi: 10.1038/s42003-022-03916-6 (PMC9464190; doi:10.1038/s42003-022-03916-6)
Supplement: Supplementary file 3 — Description of Additional Supplementary Files [file 42003_2022_3916_MOESM3_ESM.pdf]

Description of Additional Supplementary Files

File name: Supplementary Data 1
Description: The source data behind the graphs in the manuscript.

File name: Supplementary Movie 1
Description: Dr-Vsp is localized at the endosomal membranes of early endosomes. MDCKII cells co-expressing Dr-Vsp-mCherry and Rab5-EGFP for early endosomes. Timelapse images were acquired from the same samples as presented in Figure 4bI. Magenta, Dr-Vsp. Green, Rab5. Scale bar = 20 µm.

File name: Supplementary Movie 2
Description: Dr-Vsp is localized at the endosomal membranes of recycling endosomes. MDCKII cells co-expressing DrVsp-mCherry and Rab11-EGFP for recycling endosomes. Time-lapse images were acquired from the same samples as presented in Figure 4bII. Magenta, Dr-Vsp. Green, Rab11. Scale bar = 20 µm.
