# Supplementary material for: Voltage-sensing phosphatase (Vsp) regulates endocytosis-dependent nutrient absorption in chordate enterocytes
Source: Commun Biol. 2022 Sep 10;5:948. doi: 10.1038/s42003-022-03916-6 (PMC9464190; doi:10.1038/s42003-022-03916-6)
Supplement: Supplementary file 7 — Reporting Summary [file 42003_2022_3916_MOESM7_ESM.pdf]

nature portfolio

Corresponding author(s): Yasushi Okamura, Takafumi Kawai

Last updated by author(s): Aug 9, 2022

Reporting Summary

Nature Portfolio wishes to improve the reproducibility of the work that we publish. This form provides structure for consistency and transparency in reporting. For further information on Nature Portfolio policies, see our Editorial Policies and the Editorial Policy Checklist.

Statistics

For all statistical analyses, confirm that the following items are present in the figure legend, table legend, main text, or Methods section.

n/a	Confirmed	
☐	☒	The exact sample size (*n*) for each experimental group/condition, given as a discrete number and unit of measurement
☐	☒	A statement on whether measurements were taken from distinct samples or whether the same sample was measured repeatedly
☐	☒	The statistical test(s) used AND whether they are one- or two-sided<br>*Only common tests should be described solely by name; describe more complex techniques in the Methods section.*
☐	☒	A description of all covariates tested
☐	☒	A description of any assumptions or corrections, such as tests of normality and adjustment for multiple comparisons
☐	☒	A full description of the statistical parameters including central tendency (e.g. means) or other basic estimates (e.g. regression coefficient) AND variation (e.g. standard deviation) or associated estimates of uncertainty (e.g. confidence intervals)
☐	☒	For null hypothesis testing, the test statistic (e.g. F, t, r) with confidence intervals, effect sizes, degrees of freedom and P value noted<br>*Give P values as exact values whenever suitable.*
☒	☐	For Bayesian analysis, information on the choice of priors and Markov chain Monte Carlo settings
☒	☐	For hierarchical and complex designs, identification of the appropriate level for tests and full reporting of outcomes
☒	☐	Estimates of effect sizes (e.g. Cohen's *d*, Pearson's *r*), indicating how they were calculated

Our web collection on statistics for biologists contains articles on many of the points above.

Software and code

Policy information about availability of computer code

Data collection	*Provide a description of all commercial, open source and custom code used to collect the data in this study, specifying the version used OR state that no software was used.*
Data analysis	ImageJ (image processing), GraphPad Prism 8 (graph and statistical analysis), iDEP v0.92 (RNA-seq analysis), COBALT and MEGAX (multiple amino acid alignments and molecular phylogenetic tree)

For manuscripts utilizing custom algorithms or software that are central to the research but not yet described in published literature, software must be made available to editors and reviewers. We strongly encourage code deposition in a community repository (e.g. GitHub). See the Nature Portfolio guidelines for submitting code & software for further information.

Data

Policy information about availability of data

All manuscripts must include a data availability statement. This statement should provide the following information, where applicable:

- Accession codes, unique identifiers, or web links for publicly available datasets
- A description of any restrictions on data availability
- For clinical datasets or third party data, please ensure that the statement adheres to our policy

Data generated or analyzed in the present study are included in this published article and its supplementary information files. Key reagents and resources are listed in Supplementary Table 3. Further information and requests should be directed to and will be fulfilled by the corresponding author.

Human research participants

Policy information about studies involving human research participants and Sex and Gender in Research.

Reporting on sex and gender	*Use the terms sex (biological attribute) and gender (shaped by social and cultural circumstances) carefully in order to avoid confusing both terms. Indicate if findings apply to only one sex or gender; describe whether sex and gender were considered in study design whether sex and/or gender was determined based on self-reporting or assigned and methods used. Provide in the source data disaggregated sex and gender data where this information has been collected, and consent has been obtained for sharing of individual-level data; provide overall numbers in this Reporting Summary. Please state if this information has not been collected. Report sex- and gender-based analyses where performed, justify reasons for lack of sex- and gender-based analysis.*
Population characteristics	*Describe the covariate-relevant population characteristics of the human research participants (e.g. age, genotypic information, past and current diagnosis and treatment categories). If you filled out the behavioural & social sciences study design questions and have nothing to add here, write "See above."*
Recruitment	*Describe how participants were recruited. Outline any potential self-selection bias or other biases that may be present and how these are likely to impact results.*
Ethics oversight	*Identify the organization(s) that approved the study protocol.*

Note that full information on the approval of the study protocol must also be provided in the manuscript.

Field-specific reporting

Please select the one below that is the best fit for your research. If you are not sure, read the appropriate sections before making your selection.

☒ Life sciences ☐ Behavioural & social sciences ☐ Ecological, evolutionary & environmental sciences

For a reference copy of the document with all sections, see nature.com/documents/nr-reporting-summary-flat.pdf

Life sciences study design

All studies must disclose on these points even when the disclosure is negative.

Sample size	No sample size calculation was performed, but we set the enough sample sizes to detect the significant difference based on the variability. The sample size used for each experiment are described in the figure legends.
Data exclusions	No data was excluded from the analyses.
Replication	Experiments were independently repeated, and all replication attempts were successful. Definitions of replicates and statistical analysis are reported in the figure legends.
Randomization	All samples were randomized within the same genotype at the time of collection.
Blinding	Researchers were not blinded to the samples' genotypes during experiments and outcome assessments.

Reporting for specific materials, systems and methods

We require information from authors about some types of materials, experimental systems and methods used in many studies. Here, indicate whether each material, system or method listed is relevant to your study. If you are not sure if a list item applies to your research, read the appropriate section before selecting a response.

Materials & experimental systems

n/a	Involved in the study	
☐	☒	Antibodies
☐	☒	Eukaryotic cell lines
☒	☐	Palaeontology and archaeology
☐	☒	Animals and other organisms
☒	☐	Clinical data
☒	☐	Dual use research of concern

Methods

n/a	Involved in the study	
☒	☐	ChIP-seq
☒	☐	Flow cytometry
☒	☐	MRI-based neuroimaging

Antibodies

Antibodies used	- Mouse monoclonal Anti-Zebrafish VSP/TPTE (clone N432/21; UC Davis/NIH NeuroMab Facility; Cat# 73-485; RRID: AB_2716253)<br>- Rabbit monoclonal Anti-Sodium Potassium ATPase (Abcam; Cat# ab76020; RRID: AB_1310695)<br>- Acti-stain™ 488 Phalloidin (Cytoskeleton, Inc.;Cat# PHDG1)<br>- Alexa Fluor™ 594 Phalloidin (Invitrogen; Cat# A12381; RRID: AB_2315633)
Validation	Antibodies were validated by manufacturers. For in-house validation, the negative signal of Anti-Zebrafish VSP/TPTE was confirmed in Dr-VSP-deficient zebrafish.

Eukaryotic cell lines

Policy information about cell lines and Sex and Gender in Research

Cell line source(s)	MDCK type 2 cells were purchased from ATCC (CRL-2936).
Authentication	The authenticity of the cells was provided by the American Type Culture Collection upon purchase.
Mycoplasma contamination	Mycoplasma-free cultures were used for the experiments.
Commonly misidentified lines (See ICLAC register)	No commonly misidentified cell lines were used in this study.

Animals and other research organisms

Policy information about studies involving animals; ARRIVE guidelines recommended for reporting animal research, and Sex and Gender in Research

Laboratory animals	- Zebrafish (Danio rerio) embryos, larvae, and adults (RIKEN wild-type, Tg(vsp:EGFP) transgenic, and Dr-VSP-deficient strains).<br>- Ciona intestinalis Type A (C. robusta) juveniles and young adults (wild-type).
Wild animals	The study did not involve wild animals.
Reporting on sex	The study was conducted with no consideration for sex information.
Field-collected samples	The study did not involve samples collected from the field.
Ethics oversight	Protocols used for the animal experiments in this study were approved by the Animal Research Committee of Osaka University, Japan. All procedures were conducted in accordance with the regulation of the Animal Care Facility, Center for Medical and Translational Research (CoMIT) at Osaka University, Japan.

Note that full information on the approval of the study protocol must also be provided in the manuscript.
